# Information preferences of patients with chronic blood cancer: A qualitative investigation

**DOI:** 10.1371/journal.pone.0293772

**Published:** 2024-08-20

**Authors:** Debra A. Howell, Dorothy McCaughan, Alexandra Smith, Russell Patmore, Eve Roman

**Affiliations:** 1 Department of Health Sciences, Epidemiology & Cancer Statistics Group, University of York, York, United Kingdom; 2 Queens Centre for Oncology and Haematology, Castle Hill Hospital, Cottingham, United Kingdom; University of Macerata: Universita degli Studi di Macerata, ITALY

## Abstract

**Background:**

Haematological malignancies (blood cancers) often follow chronic trajectories that can span many months or years. Management of these diseases typically involves periods of ‘Watch & Wait’, whereby patients are monitored by the hospital and only treated at progression, if or when this occurs, which might be never or on multiple occasions. Such remitting-relapsing pathways are unpredictable and can cause anxiety and distress. This study aimed to generate evidence about information preferences, with a view to underpinning future patient-facing resources; potentially mitigating psycho-social difficulties and promoting effective shared decision-making.

**Methods:**

A qualitative study was conducted, set within a UK population-based cohort of patients with haematological malignancies. Sampling was purposive, based on age (initially around the median age of diagnosis) and disease subtype (chronic lymphocytic leukaemia, follicular lymphoma, marginal zone lymphoma and myeloma); and in-depth interviews took place with 35 patients (10 with relatives). Analysis drew on qualitative description and thematic content analysis and included critical reading and annotation of transcripts, identification of common and rare phenomena, generation of codes and coding of material, and theme development.

**Results:**

Patients discussed their preferences and experiences at length and rich data were generated from diagnosis onwards, across diagnostic subtypes. The overarching theme identified was ‘Variations in preferences’ with needs seen to differ from person to person; as well as changing over time for individuals. Five sub-themes were identified: 1) To know or not to know? 2) Needs are dynamic; 3) The polarising issue of prognosis; 4) Preferred sources; and 5) Differences in content, depth and presentation.

**Conclusions:**

Varied, dynamic information preferences indicate that resources should be developed in a way that provides maximum choice, enabling patients to select relevant material at different time-points on their trajectory. The development of blood cancer subtype-specific “real-world clinical scenarios” could improve patient experiences and inform shared decision-making.

## 1. Background

Arising in blood and lymph forming tissues, haematological malignancies (leukaemias, lymphomas, and myelomas) collectively comprise the fifth most common cancer in developed countries [1]. With diverse aetiologies, treatments and outcomes, numerous subtypes are currently recognized by the World Health Organization [2,3]. Although incidence of blood cancer is stable in the UK, prevalence is increasing due to population ageing and new treatments. Around 60% of these diseases are incurable [4], and unlike aggressive subtypes (potentially curable with intensive, often toxic treatment) typically follow similar pathways to those of chronic diseases [5,6], with varying periods of stability and progression; although some may be associated with rapid relapse and demise.

Management of chronic, indolent blood cancers typically involves periods of ‘Watch and Wait’ (W&W), whereby patients are monitored at regular intervals and only treated if and when progression occurs, which might be never, or on multiple occasions over many months or years. The decision to commence treatment is determined by cancer stage, speed of progression, symptom burden, performance status and comorbidity, as well as previous treatment lines and tolerance; and it aims to regain remission, reduce symptom burden and prolong life. Outcomes differ by subtype, as is reflected in five-year net survival estimates, which are much poorer for myeloma (48%) than chronic lymphocytic leukaemia (CLL), follicular lymphoma (FL) and marginal zone lymphoma (MZL) (86%, 88% and 80% respectively): https://hmrn.org/statistics/survival).

While some patients are reassured that their cancer is chronic, others struggle with the uncertainty this entails [7], which may result in prolonged emotional and psychological difficulties [8,9], including overwhelming feelings of anxiety, distress, turmoil, panic and isolation [10–12]. Such issues may be exacerbated by infrequent hospital appointments related to the cancer’s chronicity, meaning opportunities for discussion and reassurance are limited [13]. Importantly, as patients with very indolent subtypes can survive for many years and eventually die from other causes, this means they may also live day to day with enduring and significant psychological and emotional difficulties.

Published in 2019, the UK’s NHS Long Term Plan [14] aimed to empower people and transform health experiences for individuals with complex conditions. A key goal was for patients to have access to quality, trustworthy information to facilitate informed decision-making about their care. This is important in the context of chronic blood cancers as multiple treatment options may be available, of which patients must be aware if they are to effectively participate in the decision-making process [15]. Furthermore, patients whose information needs are satisfied report better quality of life, and less depression and anxiety than those with outstanding requirements [16,17], possibly because they understand potential future events better, so can make choices more effectively.

Research examining information-related issues in people with chronic blood cancer reports that this group tends to receive inferior information that is sparse and inconsistent [18], as well as suboptimal and insufficient [19]. They are often said to be dissatisfied with the material provided, and note scope for improvement [19–21]. Unsurprisingly, they are also said to have have poorer diagnostic understanding than patients with other malignancies, with only 52% of people with myeloma saying they understood their diagnosis, compared to 73% of all cancers [22]. Additionally, despite acute and chronic blood cancers differing markedly, many exisiting studies combines subtypes (e.g. all haematological malignancies or all lymphomas), thus losing specificity. This present study aimed to provide evidence about information preferences to underpin future resources and visualisations, with a view to mitigating psycho-social issues and promoting effective decision-making.

## 2. Methods

Qualitative methods were used and are described in accordance with the Consolidation for Reporting Qualitative Research (COREQ) [23] (S1 Table). The study was set within a population-based cohort of people with blood cancer, initiated in 2004 in England to inform research and clinical practice [4,24,25]. Haematology teams across 14 hospitals in the area provide care according to national guidelines, and people affected by blood cancer prioritised and co-developed the study. Ethical approval was granted from Leeds West (04/Q1205/69) and London, City and East (16/LO/0740). Patients who agree to further contact via the cohort can be approached for further research purposes, such as that described here.

Individuals from the cohort identified as ‘information-rich’ [26] (i.e. they had experience of CLL, FL, MZL or myeloma diagnosis and single or multiple episodes of treatment/observation, so could provide data relevant to this research) were identified via purposive sampling. Patients around the median diagnostic age by subtype were first targeted, with variation by socio-economic area of residence and time since diagnosis; and differing age-groups later added to capture more diverse experiences.

Selected individuals were sent an information sheet and asked to contact the study team if they wanted to take part. They were also given the option of inviting a relative or other person to join them in the interview, if they wished. From February to October 2019, and with informed, written consent (verbal from relatives), semi-structured in-depth interviews were conducted by a single researcher (DM) to collect data. Patients were asked to explain their experiences in their own words, with a broad topic guide (developed with expert clinicians from the study team, patients and relatives: S2 Table), used to ensure all areas of interest were covered. Each interview lasted 60–90 minutes and was audio-recorded, transcribed verbatim by hand by an independent company, and anonymised. Recruitment was guided by data saturation [27].

Systematic data analysis was conducted collegially, by two members of the team (DH and DM) and drew on qualitative description, a minimally theorised method that is relevant to clinicians and policy makers [28], and thematic content analysis [29,30]. Initially, the researchers familiarised themselves with the data by critical reading, re-reading and annotating the transcripts by hand, whilst attempting to interpret accounts. An iterative process was utilised to generate meaningful codes, discuss differences in interpretation, and make any necessary revisions. Similar codes were then identified, and reviewed within a thematic map before the themes were finalised, defined and named. Verbatim quotations are presented in the Results, to enable the interviewees to ‘speak for themselves’ about their experiences, and to support the themes, which provide insight into ‘what is going on’ [29,30].

## 3. Results

**Fig 1:**
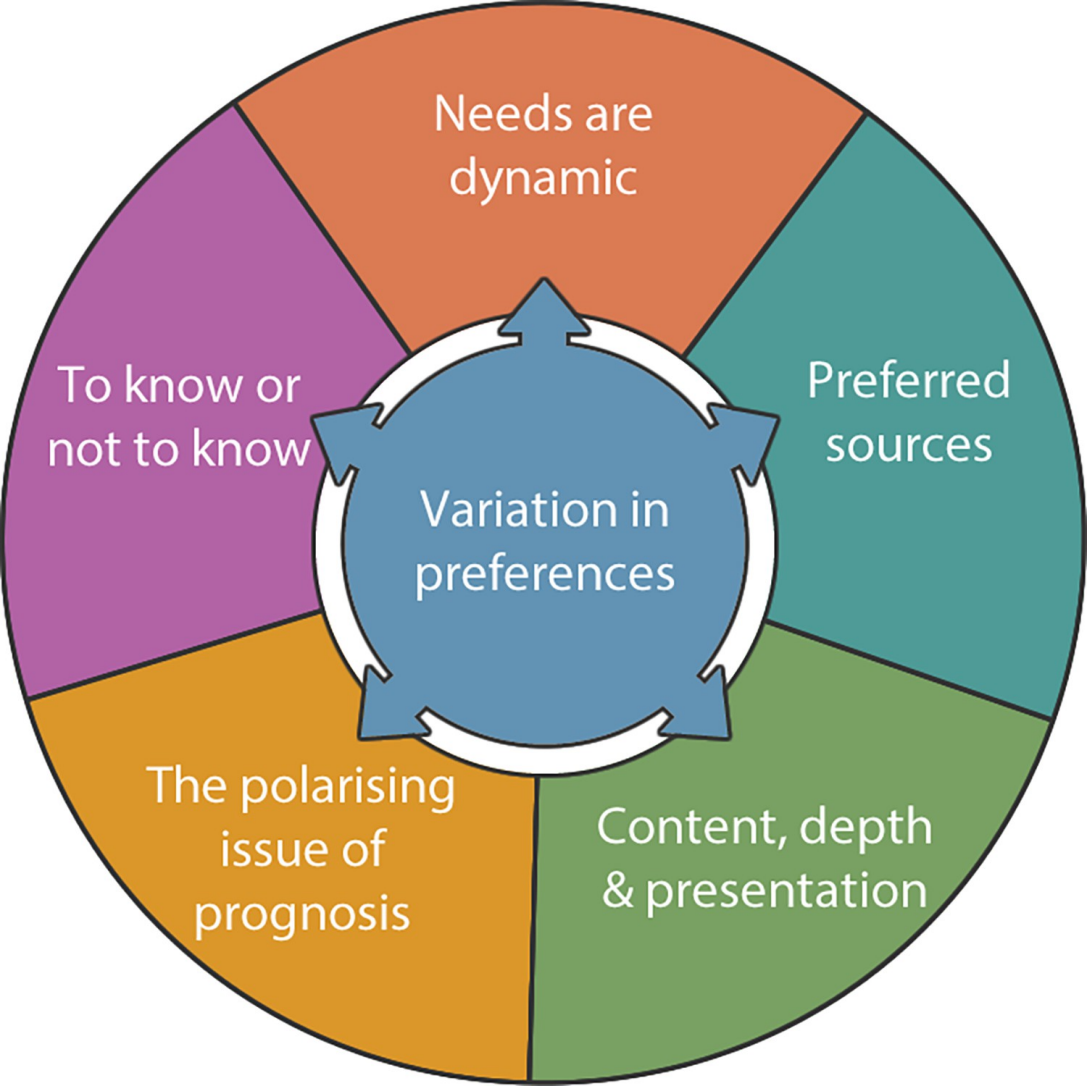
Overview of themes.

Thirty-five patients were interviewed (ten with a relative present) who had experienced varied pathways, determined by their diagnosis and pattern of progression: seven began and stayed on W&W; the remainder receiving at least one treatment, with six having multiple lines of chemotherapy prior to stem cell transplant (SCT): (S3 Table). The overarching theme identified was: ‘Variation in preferences’ with needs differing person to person, and for the same person over time. This is captured in five interrelated themes: 1) To know or not to know? 2) Needs are dynamic; 3) The polarising issue of prognosis; 4) Preferred sources; and 5) Differences in content, depth and presentation (Fig 1). Sub-themes are described below with quotations, linked to participants (P1 = Patient 1; P1R = P1’s relative).

### Theme 1: To know or not to know?

Variability was noted in patients’ desire for information about their cancer, with many being active; P18 being ‘*someone who wants to understand* things’; and P23 saying ‘*as much information as possible is a good thing…it’s about you and it’s important that you know…*’; and P11 and his wife considering this as: ‘*trying to arm ourselves with information’*.

Others felt unable to receive information or chose to avoid it altogether, particularly at diagnosis, with P33 saying that after being given ‘*paperwork’* at this time, she was ‘*so emotional’* that she said *‘I can’t read any of this stuff’*. Similarly, P35 considered the written material given to her as premature: *‘I could hardly bear to look at it really’*; as did P4 who was told her prognosis by a nurse at diagnosis (*“some people live 5 years with it”*) and said ‘*you’ve just been told you’ve got cancer and she’s saying you might only live for 5 years…I didn’t need to hear that one when I’d just been told I’d got cancer’*.

Patients who adopted a more passive stance were less likely to request information, instead choosing to wait for it to be given, such as P30 who had been told little about the potential course of his disease: ‘*if anybody gives me any inkling into what’s happening*, *I’ll listen by all means*, *but nobody has mentioned owt [sic] yet*’. Similarly, P5 said she had not been told her cancer stage, but: *‘then I haven’t asked the question either’*. Sometimes not asking was due to lack of understanding: *‘the specialist…(gave test result) which meant nothing to me…I never asked*. *I said “Okay*, *I’ll just leave it in your hands”‘* (P31).

Choosing not to receive information was perceived by some as a way of relinquishing responsibility, with P22R taking the view that *‘ignorance is bliss’*, and P19 describing this was a way of ‘*shutting things out’* and a *‘coping mechanism’*; whilst also saying she *‘didn’t want to know’* because she was too ill at the time. Patients taking this approach tended to rely on the expertise of clinical staff, P10 saying *‘I put my absolute trust in (Healthcare Professionals*: *HCPs)*, and P21 noting a willingness to *‘put (him)self in (HCPs’) hands and just run with them’*. Others relied on the relatives and friends, who were described as information brokers on the patient’s behalf, with P27R saying she read material for her husband as he was not ‘*desperate’* to see it; and P35’s friend calling a myeloma helpline on her behalf.

Relatives were said to have their own information needs (P28) that could differ from those of patients, with preferences seen to vary within families, P17 saying she liked to read and research about her lymphoma, whereas her husband was *‘the sort of person that just wants to get on with it*, *rather than know all the details…(he’s more) “you can tell me afterwards”‘*. Some relatives (e.g. P7R) wanted information for themselves about, for example, about sources of support.

### Theme 2: Needs are dynamic

Variation was evident in preferences for ‘when’ individuals wanted to receive information, with this seen to shift over time. Some wanted to know immediately, P34 saying *‘the first thing I did when I was told it was probably lymphoma*, *was go and find out as much as I could*’. Others were only able, or prepared, to know later, including P19: *‘I didn’t want to know for some reason*…*I think even after my chemo…nearly a year I think*. *I would start to do a bit of research and then I’d see something I didn’t want to know*, *so I would shelve it for a while*, *and then I’d pick it up again but it was probably a year’*.

Further elucidating the ‘process’ of gaining information, P4 described being unable to formulate questions at diagnosis, then searching the internet so she would know what to ask next time: *‘So when I went back (to clinic)…I had lots of questions…I think it was only over time…probably a year*, *if not longer*, *before I fully understood what I’d got and what it was doing to your body*, *or could do*.*’*

Some only desired information up until the point where they became familiar with their diagnosis. P21 for example said that initially *‘you want to know all about it… (then) you don’t want to know any more…you just get on with (it)’*; and P3 noted being ‘*a bit obsessed’* with his blood results at first, then satisfied for his consultant to convey these. Similarly, P34 reached a point when he decided to stop seeking information: ‘*I know as much as I can*, *but I don’t want to get too obsessed with it*. *I don’t really look…I don’t bother…I’ve got enough now*. *I don’t really want to read any more about lymphoma…there’s no point in knowing every single thing about it’*. P11 also said that he had not sought new material for *‘a good few years’*, nor had P35.

Preferences appeared to change after more was learnt about the cancer diagnosis, which seemed to enable patients to set their own limits. At diagnosis for example, P13 had been interested in her prognosis, but after reading a booklet that said: *“you can live with this condition for 10*, *15*, *20 years”*, decided *‘“that’s fine” and put the booklet away’;* opting only to refer to it if her disease progressed. Similarly, P32 communicated her needs to medical staff, telling HCPs: *‘I don’t want to know any more’* after being informed she would *‘have a normal lifespan’*.

Some preferences changed at progression after which more information was sought (P3, P13, P20); with P3 describing searching the internet *‘a bit more often since I came out of remission*, *probably about 3 times a week*, *3 or 4 times a week I would say…sometimes more*, *sometimes less…I might spend hours on it…other times just a quick glance…’*. This was more apparent if treatment decisions were required, P35 saying ‘*before*, *I didn’t have any choice (about treatment); this time (post-relapse) I am going to have options and I’m going to find that really hard because I’ve got to find out all about it and do the research’*.

A common theme in the face of uncertain pathways was the need for information about the present, but not the future, which enabled some to continue their lives without being overwhelmed. The juxtaposition between the present situation and potential future events led P18 to say he preferred information *‘stage by stage…as needed…without overloading you…as too much detail of what was down the line (would be) too much to take on’*. Similarly, P35 said: *‘I didn’t want to know everything straightaway*, *and they don’t tell you everything straightaway…they keep things back*, *and I can really understand why*, *because like if they’d said*, *“straight after that (treatment)*, *you’ll be having a stem cell transplant*, *and that means this*…*”*, *your head would just explode’*. In further examples, P34 chose to put aside knowledge that progression would occur until necessary: ‘*I just think that’s all later…it’s down the line*, *or it may never come back*, *who knows…doctors haven’t told me’*; and, P17 said that although not ‘*blinkered’*, she only wanted to know about treatment later, to avoid premature worry: *‘I’d rather think (about) it when it happens*, *and if it happens’*.

### Theme 3: The polarising issue of prognosis

Prognostic information was particularly polarising, with some participants strongly against hearing this, perhaps due to the uncertain nature of their cancer, instead favouring a more philosophical approach. P30 for example said he was ‘*not particularly bothered’*, taking the view that *‘if it comes*, *it comes…I can’t do owt [sic] about it’*; while P3 noted the difficulty of knowing the right time to ask.

Some patients were afraid to hear about the future in case: *‘it turns out to be bad…(and) you are in one of the “outlook not so good” groups*’ (P3); with P35 saying *‘I’ve never dared ask how long I might live…it’s just too big…if I could know it*, *I don’t know if I’d want to’*. Similarly, although aware of his limited life-expectancy, P31 chose not to explore this: ‘*No*, *we haven’t really had any information…I mean it’s a bit obvious that at some point [myeloma is] going to kill me*, *you know what I mean…you don’t need to be a rocket scientist do you*. *But they’re looking after me and I’m very happy with what they’re doing*, *so I don’t worry’*. P29 didn’t subscribe to the *‘how long have I got thing (due to) exceptions*: *either they died next day*, *or “I was given a year to live and now I’ve been alive 3 years”‘*.

A desire to be fully informed about prognosis was also noted, with P25 wanting to know *‘what is the likelihood of it coming back…what’s the odds…how many people live to a ripe old age and die of something else’*. Some predominantly wanted to know about prognosis above all else, even at diagnosis, with P28 saying that although *‘scary (he) instantly (searched online)*, *like how long am I going to live (and) made (it his) business’*. P13 said she *‘needed to know in my own mind what might come’*. Some searched for prognostic information online, such as P3, who said he *‘definitely’* wanted to know this, but had *‘never really discussed it’* with his consultant.

Family members could have a similar mindset, with P20R (CLL) saying she had wanted to know *‘immediately*, *what’s the prognosis*, *what’s the long-term*?*’*. Similarly, P6R said *‘it was not a nice position to be in*, *thinking well*, *there is this disease and we don’t really know how this is going to play out…’*; and P27R wanted to know ‘*what we were trying to achieve’*.

Patients appeared aware of the varied preferences for prognostic knowledge, P2 saying *‘the thing is giving people enough information for what they need…a lot of people would say “I don’t want to know*, *I don’t want to know”*, *just let them get on with it*. *Whereas for me*, *I wanted to know what is wrong with me*, *what the outcome would be’*; and P3 noting: *‘everyone is different…that’s what I learned*. *You have to decide what suits you*, *what you feel comfortable with*.*’*

A ‘*direct*…*straight’* approach with specific terminology was preferred by some, rather than ‘*very vague answers’* (P2) and use of ambiguous phrases and terms, such as *‘you can survive with this for a while’* (P3). P21R said *‘we wouldn’t mind if they said we’ve only dealt with two people in this hospital and they didn’t last longer than this length’* rather than being told *‘“people in this situation have responded well to treatment”; what does this mean*?*’*. Interestingly, prognostic knowledge was said to facilitate decision-making, for example when comparing survival with or without the recommended treatment (P1, P7, P8).

Some people wanted personalised prognostic knowledge, with P28 saying his HCPs *‘wouldn’t talk about ‘my’ condition…they would talk about the population as a whole…that fifty percent figure… fifty percent chance of surviving 5 years is an easy thing to remember… (but) most people don’t get myeloma at 59*, *they get it in their mid-70s…their life expectancy is very likely less than mine*, *because I’ve always been healthy and fit’*. Similarly, P3 said he would not ‘*stress out…as the whole population is not me’*; and P5 suggested such information ‘*doesn’t necessarily mean it is your path*’, and that she did not want to live life ‘*around ifs and buts*’.

### Theme 4: Preferred information sources

Preferred information sources differed, although many interviewees wanted material ‘*verbally’* (21R, P2, P4) from clinical staff: *‘somebody to talk to me…face to face’* (P25); *‘I’m not a reader*. *I like face to face’* (P33). One-to-one consultations were also preferred, as patients wanted to express views, ask questions and ensure they understood answers (P12, P14, P29).

Many others appreciated written material, including leaflets and booklets, particularly if they like to ‘*read and research things*’ (P19) or had memory problems ‘*I can always go back to it…refer to it’* (P11). Others said they preferred written material to explanations shared on the computer screen with their consultant (P7, P12). P25 differed, however, saying written material would not suit people who struggle to read: ‘*just as well I aren’t dyslexic*’, or who were particularly anxious, as she was, resulting in her reading the booklet time and again.

Charity magazines were popular, particularly if they contained articles on new treatments and ‘real life’ stories. These were described by some as *‘absolutely fantastic…really useful’* (P18); and *‘helpful and informative…not too much waffle’* (P13). They were also said to *‘cater for a spectrum of people’* (P5), via simple to in-depth explanations and the clear presentation of complex information (P18).

Information booklets, often provided by Charities, were perceived as good at *‘factual things’* but negligent of *‘the emotional side of it’* (P25), described by P28 as: *‘all the other side of being in hospital’*. They could also be perceived as overly complex: *‘some of the words*, *you can’t understand…too many syllables’* (P30). Similarly, P31 said his booklet was ‘*a bit too high a level’*; although his wife thought it *‘clear and did its job’*, highlighting variability within families. Compared to the internet, however, such booklets were seen as valid, reliable and trustworthy because *‘somebody has cleared…and standardised (them)’* (P26).

Booklets were said to contain general information about more common conditions, but less on rarer subtypes (P5, P21R), and in this context lacked the *‘finer details’* (P15). This led P9 to say ‘*some of these leaflets are misleading and it caused me some anxiety*. *(Nurse) said “this does not affect you” (about one section)*, *but reading the leaflets I didn’t realise that…; face to face you can say “stop*, *what do you mean by that*?*”‘*

A common preference was to receive both verbal and written information (e.g. P2, P4, P17). P15 liked *‘word of mouth’* and a leaflet, as did P12 who said he wanted to talk, but also *‘walk away with something in your hand…I think that would be quite important*, *something you could go away with and digest’*. Similarly, P11 said that everything he was told by the nurse specialist was ‘*backed up’* by written material to read later. Others preferred verbal and computer information, P34 saying *‘I like it when they do (screen sharing)’* as he could see the graph of *‘where I’m at*, *what’s going on*, *how its improved or gone down’*; as was echoed by P3.

The internet was often discussed as an information source although views on its utility and validity varied. Certain websites were perceived as ‘*brilliant’* (P6R) or ‘*excellent’* (P13). YouTube videos were said to *‘speak’* to some in a way they liked, particularly as these could play in the background or be re-watched (P33). Some websites were considered *‘old hat’* (P33) or too broad in the information they provided, with an example being prognostic estimates that ranged from several to 20 years (P34).

The internet was also described as ‘*terrifying’* (P35); a place for *‘frightening yourself to death’* (P25, echoed by P31, P31R, P33); and downcast: *‘always seems to give the worst scenario’* (P32); ‘*makes you think you’re going to die of everything’* (P34); ‘*you can have everything from A to Z’* (P4). Interviewees described not knowing which sites were reliable or accurate (P3, P13), and needing to ‘*cherry pick’* to avoid those that were ‘*dreadful… scary…and (may not) give you the information you trust or want to hear’* (P14). Some patients were advised by consultants against online searches: *‘whatever you do*, *just don’t pick up CLL on the internet*, *just don’t go there’* (P13). P35 suggested that a database containing collated, reliable information would ‘*save you from looking at loads of stuff that you don’t need to see or you’re not sure what it is’*.

P20 noted that skills were required to carry out ‘*intelligent’* online searches, for ‘*authoritative’* information (P18), which some said they lacked: *‘not very good with technology’* (P1); or did not have any desire to pursue: ‘*I’m not an internet person* (P15). Some relatives conducted searches on the patient’s behalf (e.g. P20), because they were *‘internet savvy’*, had better concentration or memory (P16); or if the patient could not perform this task themselves (e.g. P15, with poor vision). Conveying the unwanted consequences of online searching, P21R was home alone when she found her father may only have *‘six months to live’*, and found herself *‘in bits…it was hideous’*.

### Theme 5: Differences in content, depth and presentation

Preferences for information depth and content differed greatly, highlighting the delicate balance between having sufficient information that is not too ‘*vague*, *meaningless’* (P21R), yet not too much detail, which could be *‘overwhelming’* (P33). Less complex or exhaustive material was preferred by some: *‘I don’t want to go into details’* (P10); *‘(not a) huge amount of technical information’* (P6R). Similarly, P13 wanted: *‘little bits of information at a time…without a lot of detail’*; as did P29 and P35. Some wanted general information: ‘*what is this going to do to my life*?’ (P26); *‘(I) just wanted to know how (treatment) would affect me…if I’d be ill*, *what I was going to be like’* (P35). This was the case for P27R, who described relying on the specialist nurse to ‘*summarise’* progress rather than give a detailed account. Fear seemed to impact this, with P35 saying: *‘there are some people who want to know every detail about every treatment and how it affects them*. *I’m a bit scared to do that…’*.

Some patients said they wanted information in great detail, however, with P23 considering *‘as much information as possible is a good thing’* (echoed by P2, P3, R6, P12), with P32 saying the more ‘*firepower’* the better. Others described wanting precise information, with P34 (FL) noting: *‘I have asked specifically*, *how many little tumours and where is it*, *and I’ve been told the size of them*, *and where they are in the body and stuff like that’*. Preferences for detailed information were not always met, with P21 describing how prognostic questions elicited *‘euphemisms’* and imprecise language; while P32R said ‘*(HCPs) were a bit vague*. *They wouldn’t commit themselves to anything’*; and P2 noted ‘*I have asked specific questions and not been given the answers that would help me’*. Similarly, 6R felt clinicians could underestimate what patients wanted to know: *‘assum(ing) Mr and Mrs Average isn’t quite capable of understanding’*.

Regarding presentation, some interviewees preferred to receive information in diagrams rather than words (P6), with P13 being ‘*more of a visual person’* due to being *‘slightly dyslexic’* and liking ‘*family tree’* type images; while P18 wanted material shared by the haematologist in *‘graphs*, *charts*, *numbers…’*, as did P1, P6R and P34. ‘Apps’ were considered a *‘no-no’* for those without ‘*technical skills’* (P1), but some liked to view their electronic medical record prior to clinic: *‘platelets*, *white (blood cells)…haemoglobin… that extra information*, *for me*, *is very valuable… I can (ask consultant) “what’s this about”*, *“why’s that gone up”‘* (P28).

Perceptions of the value of numerical information varied, P24 saying he *‘always likes to know the figures’*; and P3 finding statistics useful, although P22R believed these could cause unnecessary worry: *‘I wouldn’t look at statistical information…because I think it can be frightening…if they say “Oh*, *well 10% do this and 5% live that long”*, *I don’t want to know because I think you are an individual’*. P11 said *‘(You) can’t tell with statistics if you will be the 1 in 100’*, while P16 noted that these can be ‘*read in different ways (and are) OK in an analytical world…but you’ve got this human nature side’*. P6 said *‘I don’t mind percentages’*, but others disagreed, preferring absolute figures, or use of phrases, such as ‘*I would say ‘possibility’ might be best…rather than a 30% chance’* (P14).

## 4. Discussion

The most striking finding and overarching theme from our study was the variable, dynamic information preferences of individuals with chronic blood cancer and their families. This included if, and what people wanted (or were ready) to know, when they wanted to know it, in what format, and how this changed over time. Importantly, the ability to meet such differing requirements underpins the extent to which information needs are met, thus it has a crucial role in allaying anxiety and promoting quality of life [16,17], without which dissatisfaction and poor disease understanding is likely to persist [20–22]. Furthermore, the acquisition of adequate information, in the preferred format, could facilitate evidence-based decision-making, as recommended in UK Health policy [14]. Modification of available material and processes may also reduce the time and effort some patients invest in gaining knowledge to apply to their own circumstances, to aid decision-making [15].

To meet preferences, a range of information of different complexity, about multiple issues, that is able to be appropriately tailored, would need to be available to access, when required; particularly for those with rarer subtypes, where material is sparse. While some requirements could be met without too much additional resource (e.g. collating reliable website links, signposting), some major challenges exist. The first is the unpredictability of blood cancers, meaning clinicians may struggle to source accurate and current information about the likelihood of progression, need for treatment, and survival [8,13]. The second relates to the limitations to available information for sharing. For example, clinical trials often include a single treatment/time-point, examined in younger, fitter people who are not representative of the general patient population; thereby limiting knowledge about treatment outcomes and progression in older age [4].

Information preferences among people with chronic blood cancer have not been specifically, or solely examined in other qualitative studies, although unmet needs and some preferences are reported within research exploring experiences more generally. For example, in the context of CLL, varied preferences for information were also noted, without jargon [20]. Among the few qualitative studies including both acute and chronic subtypes, one noted the desire for plain and simple language; clear, concise information that was relevant, individually tailored or ‘personalised’ to the patient; and realistic discussion that balanced prognosis with hope [9]. Another noted preferences for empathetic, expert, honest, direct and meaningful information that specifically related to the patient’s situation [31]. Other research did focus solely on preferences, typically within quantitative surveys, structured interviews and literature reviews, combining chronic and acute subtypes [19,32,33]. These studies concurred with aspects of our findings, including for example, variation in information preferences [33], a desire for material that was tailored to the level preferred by the patient [19], and requests for current, accessible information, and for HCPs to assess information needs and manage and tailor material accordingly [32].

Regarding information source, it was agreed that hearing directly from a clinician was preferable to written material, or talking to other patients [18,32], although this could be limited by clinical time-constraints [9,12,20] and infrequent appointments, while patients were living day to day with their chronic condition [13]. Internet use has also been reported by others with haematological [34,35] and different cancers [36], and is often considered practical and empowering, providing the knowledge required to discuss treatment options, particularly in those who are healthcare literate, and confident in interpreting complex issues and asking questions [37]. These studies do, however, also concur with our concerns about lack of IT skills and equipment, variable quality of the material available, reaching the limits of understanding, and less internet use among older age-groups (i.e. much of the blood cancer population) and those with lower levels of education. Interestingly, the cited studies also recommend the collation of reliable website addresses, and discussion about the role of the internet in decision-making and self-management [34,35].

Information needs are considered similar among patients with blood cancer and other malignancies, although there is much more data on the latter [18], who are said to want as much, and as detailed information as possible, and to hear both good and bad news; in contrast blood cancer patients who prefer general information. This may be because studies in the cited review include people with acute, imminently life threatening blood cancers, who might be overwhelmed by their diagnosis and prognosis [31]. This response has been reported in the context of aggressive subtypes such as acute myeloid leukaemia, where information avoidance is considered a mechanism for maintaining hope [38]; with the same reaction said to be seen in other cancers [39]. Indeed, our findings are likely to be more applicable to incurable conditions with remitting/relapsing pathways and dynamic information needs, than acute cancers.

## 5. Strengths and limitations

We believe this is one of few studies using qualitative in-depth interviews to explore the information preferences of patients living with incurable chronic blood cancer (CLL, FL, MZL and myeloma) every day of their lives, alongside family members. Purposive sampling promoted the inclusion of key informants, data were rich and spanned various key points on the pathway (e.g. diagnosis, progression). We recognize that our findings are unlikely to have captured the entire range of views of the population with the diseases of interest; nonetheless, they provide in-depth insights into issues relating to the information preferences considered important by patients with chronic blood cancers. In this context, rather than generalisable to the population, our findings are likely to be transferable [29] across similar healthcare settings, and to other chronic cancers and conditions. Use of qualitative description means our findings are likely to be relevant to practitioners and policy makers [28], although the experiences of those too ill to be contacted could not be captured.

### Implications for clinical practice

Our overarching theme on varied information preferences clearly highlights the importance of HCPs, patients and families discussing this issue, so that as far as possible, individual needs can be met, whilst being mindful that these will change over time. Potential future improvements include the development of novel resources such as “real-world clinical scenarios”. These might be derived from common trajectories among people with similar demographic and disease characteristics, mapped to population-based data and used in NHS settings to aid discussion with individuals and families. Although there is little research about such resources, one study reported web-based scenarios to be feasible, helpful, acceptable and safe [40], and another demonstrated the possibility of mapping individual patient pathways in great detail, and including hospital-based activities [41]. Further co-design, development and testing with relevant stakeholders (e.g. patients, relatives and clinical staff) would be required to progress this concept, which could then also be adapted for use with other conditions.

## 6. Conclusions

Varied, dynamic information preferences were identified, indicating that resources should be developed in a way that provides maximum choice, enabling patients to select relevant material at different time-points on their trajectory, according to their needs at the time. The development of blood cancer subtype-specific “real-world clinical scenarios” could improve patient experiences and inform shared decision-making.

## Supporting information

S1 Table Consolidation criteria for REporting Qualitative research (COREQ) checklist. (DOCX)

S2 Table Topic guide. (DOCX)

S3 Table Characteristics of interviewees. (DOCX)
