# Supplementary material for: Information preferences of patients with chronic blood cancer: A qualitative investigation
Source: PLoS One. 2024 Aug 20;19(8):e0293772. doi: 10.1371/journal.pone.0293772 (PMC11335164; doi:10.1371/journal.pone.0293772)
Supplement: S1 Table — (DOCX) [file pone.0293772.s001.docx]

**S1 Table: COnsolidated criteria for REporting Qualitative research (COREQ) Checklist**

| **No. Item** | **Guide questions/description** | **Page** | **Further detail** |
| --- | --- | --- | --- |
| **Domain 1: Research team and reﬂexivity** | | | |
| **Personal characteristics** | | | |
| 1. Inter viewer/facilitator | Which author/s conducted the interview or focus group? | 4 | DM |
| 2. Credentials | What were the researcher’s credentials? E.g. PhD, MD | n/a | BA, RN, MSc |
| 3. Occupation | What was their occupation at the time of the study? | n/a | Senior Research Fellow |
| 4. Gender | Was the researcher male or female? | n/a | Female |
| 5. Experience and training | What experience or training did the researcher have? | n/a | Registered Nurse; conducted many qualitative interviews. |
| **Relationship with participants** | |  |  |
| 6. Relationship established | Was a relationship established prior to study commencement? | n/a | No |
| 7. Participant knowledge of the interviewer | What did the participants know about the researcher? e.g. personal goals, reasons for doing the research | n/a | No knowledge. |
| 8. Interviewer characteristics | What characteristics were reported about the interviewer/facilitator? e.g. Bias, assumptions, reasons and interests in the research topic | n/a | See 5. |
| **Domain 2: Study design** | | | |
| **Theoretical framework** | | | |
| 9. Methodological orientation and Theory | What methodological orientation was stated to underpin the study? e.g. grounded theory, discourse analysis, ethnography, phenomenology, content analysis | 4 | Qualitative description, thematic content analysis. |
| **Participant selection** | | | |
| 10. Sampling | How were participants selected? e.g. purposive, convenience, consecutive, snowball | 4 | Purposive. |
| 11. Method of approach | How were participants approached? e.g. face-to-face, telephone, mail, email | 4 | Mail, followed up by telephone if expressed interest. |
| 12. Sample size | How many participants were in the study? | 4 | 35 (10 with a relative present) |
| 13. Non-participation | How many people refused to participate or dropped out? Reasons? | n/a | None dropped out. 20 patients did not respond to invite for reasons unknown. |
| **Setting** | | | |
| 14. Setting of data collection | Where was the data collected? e.g. home, clinic, workplace | 4 | Their own home. |
| 15. Presence of non-participants | Was anyone else present besides the participants and researchers? | 4 | Relatives were present at 10 interviews. |
| 16. Description of sample | What are the important characteristics of the sample? e.g. demographic data, date | 5 | See S3 Table, referenced on p5. |
| **Data collection** | | | |
| 17. Interview guide | Were questions, prompts, guides provided by the authors? Was it pilot tested? | 4 | See topic guide (S2 Table), referenced on p4. |
| 18. Repeat interviews | Were repeat inter views carried out? If yes, how many? | n/a | No repeat interviews were carried out. |
| 19. Audio/visual recording | Did the research use audio or visual recording to collect the data? | 4 | Audio. |
| 20. Field notes | Were ﬁeld notes made during and/or after the interview or focus group? | n/a | The interviewer made their own notes after each interview. |
| 21. Duration | What was the duration of the interviews or focus group? | 4 | 60-90 minutes |
| 22. Data saturation | Was data saturation discussed? | 4 | Yes. |
| 23. Transcripts returned | Were transcripts returned to participants for comment and/or correction? | n/a | No. |
| **Data analysis** | | | |
| 24. Number of data coders | How many data coders coded the data? | 4 | DM, DH |
| 25. Description of the coding tree | Did authors provide a description of the coding tree? | n/a | More details given in related paper – reference given. |
| 26. Derivation of themes | Were themes identified in advance or derived from the data | 4 | Derived from data. |
| 27. Software | What software, if applicable, was used to manage the data | n/a | No software was used. |
| 28. Participant checking | Did participants provide feedback on findings? | n/a | No. |
| **Reporting** | | | |
| 29. Quotations presented | Were participant quotations presented to illustrate the themes/ﬁndings? Was each quotation identiﬁed? e.g. participant number | 5-10 | Yes. Quotations are identified by participant number. |
| 30. Data and ﬁndings consistent | Was there consistency between the data presented and the ﬁndings? | 5-10 | Yes. |
| 31. Clarity of major themes | Were major themes clearly presented in the ﬁndings? | 5-10 | Yes, and a Figure of overarching themes was presented. |
| 32. Clarity of minor themes | Is there a description of diverse cases or discussion of minor themes? | 5-10 | A range of responses are considered. |

**Developed from: Tong A, Sainsbury P, Craig J. Consolidated criteria for reporting qualitative research (COREQ): a 32-item checklist for interviews and focus groups. *International Journal for Quality in Health Care*. 2007. Volume 19, Number 6: pp. 349 – 357**
