# Supplementary material for: Information preferences of patients with chronic blood cancer: A qualitative investigation
Source: PLoS One. 2024 Aug 20;19(8):e0293772. doi: 10.1371/journal.pone.0293772 (PMC11335164; doi:10.1371/journal.pone.0293772)
Supplement: S2 Table — (DOCX) [file pone.0293772.s002.docx]

| **S2 Table: Topic guide** |
| --- |
| - Tell me about your experiences of receiving information at diagnosis, relapse and treatment? *(sufficient time, clarity, amount, ability to process material, questions, topics)* - What are your thoughts on information more generally since your diagnosis?   *(timing, clarity, detail, topics, terms used)*   - Which are your preferred sources/type of information and why? - Do you feel that your information needs are met (including asking questions and receiving answers), and if so how? *(What worked well/could have been better?)* - If any, what questions remain unanswered, or do you wish you had asked? |
