# Supplementary material for: Information preferences of patients with chronic blood cancer: A qualitative investigation
Source: PLoS One. 2024 Aug 20;19(8):e0293772. doi: 10.1371/journal.pone.0293772 (PMC11335164; doi:10.1371/journal.pone.0293772)
Supplement: S3 Table — (DOCX) [file pone.0293772.s003.docx]

**S3 Table - Characteristics of interviewees**

| **ID** | **Diagnosis**^1^ | **Age range at interview (years)** | **Years since diagnosis** | **Known treatment line(s) preceding interview**^2,3^ | | | | | |
| --- | --- | --- | --- | --- | --- | --- | --- | --- | --- |
|  |  |  |  | **1** | **2** | **3** | **4** | **5** | **6** |
| P1 | CLL | 60-70 | 4 | Observation | - | - | - | - | - |
| P2 | MZL | 60-70 | 15 | Observation | Chemotx | Observation | - | - | - |
| P3 | CLL | 60-70 | 22 | Observation | Chemotx | Observation | - | - | - |
| P4 | MZL | 60-70 | 3 | Observation | Chemotx | - | - | - | - |
| P5 | MZL | 50-60 | 2 | HPE | Observation | - | - | - | - |
| P6* | CLL | 70-80 | 8 | Observation | Chemotx | Observation | - | - | - |
| P7* | CLL | 60-70 | 6 | Observation | Chemotx | Observation | - | - | - |
| P8 | FL | 70-80 | 3 | Chemotx | Radiotx | Observation | - | - | - |
| P9 | CLL | 80-90 | 5 | Observation | Chemotx | - | - | - | - |
| P10 | FL | 70-80 | 8 | Observation | Chemotx | Chemotx | Chemotx | - | - |
| P11 | Myeloma | 60-70 | 10 | Observation | Chemotx | Observation | - | - | - |
| P12 | MZL | 70-80 | 5 | Observation | Chemotx | - | - | - | - |
| P13 | CLL | 50-60 | 1 | Observation | - | - | - | - | - |
| P14 | Myeloma | 60-70 | 4 | Steroids | Radiotx | Chemotx | Chemotx | Chemotx | SCT |
| P15 | FL | 70-80 | 3 | Observation | Chemotx | - | - | - | - |
| P16 | Myeloma | 60-70 | 2 | Chemotx | Chemotx | Chemotx | SCT | Observation | - |
| P17* | FL | 60-70 | 3 | Observation | - | - | - | - | - |
| P18 | Myeloma | 60-70 | 3 | Chemotx | Chemotx | Chemotx | SCT | Observation |  |
| P19 | FL | 50-60 | 3 | Steroids | Chemotx | Chemotx | Observation | - | - |
| P20* | CLL | 70-80 | 4 | Observation | - | - | - | - | - |
| P21* | Myeloma | 70-80 | 3 | Steroids | Chemotx | Chemotx | Chemotx | SCT | - |
| P22* | CLL | 70-80 | 3 | Observation | Clinical trial | Observation | - | - | - |
| P23 | Myeloma | 60-70 | 3 | Observation | - | - | - | - | - |
| P24 | FL | 50-60 | 4 | Steroids | Chemotx | Radiotx | Observation | - | - |
| P25 | FL | 60-70 | 4 | Chemotx | Chemotx | - | - | - | - |
| P26 | Myeloma | 70-80 | 4 | Observation | - | - | - | - | - |
| P27* | CLL | 70-80 | 4 | Chemotx | Observation | - | - | - | - |
| P28 | Myeloma | 60-70 | 4 | Steroids | Chemotx | Chemotx | SCT | Clinical trial | Chemotx |
| P29 | CLL | 70-80 | 3 | Clinical trial | Observation | - | - | - | - |
| P30* | Myeloma | 70-80 | 2 | Observation | - | - | - | - | - |
| P31* | Myeloma | 70-80 | 2 | Radiotx | Steroids | Chemotx | Observation | - | - |
| P32* | MZL | 60-70 | 2 | Observation | Chemotx | Observation | - | - | - |
| P33 | Myeloma | 50-60 | 3 | Chemotx | Chemotx | SCH | Observation | - | - |
| P34 | FL | 50-60 | 4 | Steroids | Chemotx | Chemotx | Chemotx | - | - |
| P35 | Myeloma | 50-60 | 2 | Chemotx | Chemotx | Chemotx | Chemotx | SCT | Observation |

^1^ CLL – Chronic Lymphocytic Leukaemia; FL – Follicular Lymphoma; MZL – Systemic Marginal Zone Lymphoma. ^2^ Chemotx = Chemotherapy; HPE = Helicobacter pylori eradication; Radiotx = Radiotherapy; SCT = Stem cell transplant (autografts); SCH = Stem cell harvest (shown as SCT did not take place). ^3^ Does not include supportive care (e.g. blood product transfusions, plasma exchange, bisphosphonates, cell mobilization products). *Relative present at interview.
